# Stragglers on the March

**Published:** 1886-07

**Authors:** 


### STRAGGLERS IN THE MARCH.

Two French physicians, MM. Bourrof and Burfor have recently published the results of some very interesting experiments as to the effect of various drugs at a distance, on hysterical subjects, and have claimed the merit of of a new *discovery*, notwithstanding the fact that it has been known to many for years. Among others, Dr. Buchanan publicly announced this same phenomenon long ago. The "Faculty" however, always fearful of admitting the truth of anything that they themselves may not have stumbled upon in the rut of their "regular" practice, having hitherto denied the evidence of demonstrated facts, to be consistent, now declare that MM. Bourrof and Burfor are deceived. We append a short account of their experiments :—

The principal researches were conducted on two remarkable individuals. One, a young marine, who had been under treatment at the hospital at Rochefort ; the other, a woman of twenty-six years. On approaching the subject with a bottle containing a given substance, the latter exerts an influence through the glass precisely as though it had touched the body of the patient or been ingested. All this takes place without cognizance on the part of the operator or the subject of the contents of the bottle.

If the unknown substance contained in the bottle produces intoxication, we can be certain we have to do with an alcohol. But according to whether we have to do with an alcohol of wine or one obtained from cereals, the intoxication evoked will be gay or furious. On approaching the patient with a flask containing ammonia, all the phenomena of intoxication disappear at once. All narcotics cause sleep, and emetics give rise to vomiting.

In the course of an experiment conducted before several prominent medical men, the operator carried in his pocket two flasks of the same size enveloped in paper, and wishing to test the effects of cantharides, was muh astonished to see the subject begin to scratch the earth with his fingers, make a hole in the ground and endeavor to place his face in it The explanation of this was subsequently found in the fact that instead of presenting cantharides, the operator had approached the patient with a bottle containing valerian. As is well known, the effects of the latter are the same upon man as upon the cat.

The essence of bitter almonds gives rise in women to a pronounced religious hallucination. It is in truth a veritable ecstacy, lasting more than a quarter of an hour. A few seconds after the application of the substance, the eyes are elevated, the arms are raised towards heaven. Afterwards the subject prostrates herself and assumes different expressions of countenance.

Finally, the experimenters have observed other curious phenomena, for example : terror, illusion of being bitten by a viper, the delusion of being of a different age, etc Thus a woman, being convinced that she had been carried back to the age of ten years, played with a doll precisely like a child.

The editor of a medical Journal says that "the sources of error in these experiments are considerable," evidently being willing to sit in judgment upon matters which he has never thought upon or inquired into, claiming that those who have done so are no more capable of judging of their merits than one who never investigated the subject at all.

We were once called to witness a case at Auburn, N. Y., by Justice Bostwick of that place, which, we have no doubt, the same doctor would say, never existed. Mr. Bostwick came to our room and requested us to go to his house and see a little girl, nine or ten years old, in whom he had discovered a strange faculty of seeing without the use of her eyes. He tied a handkerchief over her forehead, completely blindfolding her, then took up a book and laid it on the crown of her head where she could not see it, even if she had not been blindfolded. She read at once freely from its pages. He then tried further experiments with other books with the same result. Then we tried different articles from our pockets, which were accurately described. In all this we knew she was not using her eyes, but she saw, read and described things correctly. It was not from our minds either, for we did not know the contents of the pages until after they were read. We do not now pretend to know by what power she could do these things, but we know she did them under precisely the circumstances we have stated.

